# Closing the Loop With Cortical Sensing: The Development of Adaptive Deep Brain Stimulation for Essential Tremor Using the Activa PC+S

**DOI:** 10.3389/fnins.2021.749705

**Published:** 2021-12-08

**Authors:** Tomasz M. Fra̧czek, Benjamin I. Ferleger, Timothy E. Brown, Margaret C. Thompson, Andrew J. Haddock, Brady C. Houston, Jeffrey G. Ojemann, Andrew L. Ko, Jeffrey A. Herron, Howard J. Chizeck

**Affiliations:** ^1^Neuroscience Program, University of Washington, Seattle, WA, United States; ^2^Department of Electrical and Computer Engineering, University of Washington, Seattle, WA, United States; ^3^Department of Philosophy, University of Washington, Seattle, WA, United States; ^4^Department of Neurological Surgery, University of Washington, Seattle, WA, United States

**Keywords:** essential tremor, deep brain stimulation, machine learning, motor cortex, adaptive deep brain stimulation, fully implantable

## Abstract

Deep Brain Stimulation (DBS) is an important tool in the treatment of pharmacologically resistant neurological movement disorders such as essential tremor (ET) and Parkinson's disease (PD). However, the open-loop design of current systems may be holding back the true potential of invasive neuromodulation. In the last decade we have seen an explosion of activity in the use of feedback to “close the loop” on neuromodulation in the form of adaptive DBS (aDBS) systems that can respond to the patient's therapeutic needs. In this paper we summarize the accomplishments of a 5-year study at the University of Washington in the use of neural feedback from an electrocorticography strip placed over the sensorimotor cortex. We document our progress from an initial proof of hardware all the way to a fully implanted adaptive stimulation system that leverages machine-learning approaches to simplify the programming process. In certain cases, our systems out-performed current open-loop approaches in both power consumption and symptom suppression. Throughout this effort, we collaborated with neuroethicists to capture patient experiences and take them into account whilst developing ethical aDBS approaches. Based on our results we identify several key areas for future work. “Graded” aDBS will allow the system to smoothly tune the stimulation level to symptom severity, and frequent automatic calibration of the algorithm will allow aDBS to adapt to the time-varying dynamics of the disease without additional input from a clinician. Additionally, robust computational models of the pathophysiology of ET will allow stimulation to be optimized to the nuances of an individual patient's symptoms. We also outline the unique advantages of using cortical electrodes for control and the remaining hardware limitations that need to be overcome to facilitate further development in this field. Over the course of this study we have verified the potential of fully-implanted, cortically driven aDBS as a feasibly translatable treatment for pharmacologically resistant ET.

## 1. Introduction

Essential Tremor (ET) is one of the most common neurological movement disorders. By some estimates, it affects as much as 1% of the world's adult population and up to 4.5% of the senior population to some extent (Louis and Ferreira, 2010). ET manifests itself primarily as a 2–8 Hz tremor during active motion or holding of posture. Classically, the strongest tremor is apparent in the extremities, especially the hands, but will often also be accompanied by trunk tremor (Haubenberger and Hallett, 2018). Despite its prevalence, the non-lethal nature of the disorder means that it has been understudied for many years and the pathophysiology is still poorly understood (Soto and Fasano, 2020). Once patients are diagnosed, initial treatment is usually pharmacological, but for severe, pharmacologically refractive cases DBS is a promising option (Lyons and Pahwa, 2004).

Deep brain stimulation (DBS) is a common therapy used to treat neurological disorders. It has been approved by the FDA to treat ET, Parkinson's disease (PD), dystonia, and epilepsy; and is under investigation for treatment of depression, addiction, Tourette syndrome, and many others (Lozano et al., 2019). In current clinical practice, conventional or continuous DBS (cDBS) is used in an open-loop fashion. Stimulation is configured manually by a clinician and the applied stimulation pattern is fixed (Lyons and Pahwa, 2004). Parameter tuning is a lengthy process that, even with the expertise of a neurologist, may require several visits before a satisfactory setting is found. Optimal stimulation settings are those that significantly suppress tremor, without causing intolerable side effects. The patient is provided a “patient programmer” that they can use to turn the stimulator on or off, but this control is rather coarse at best and used primarily to conserve battery at night while patients sleep. As a result, stimulation is often delivered even when it is not necessary, which may unnecessarily increase exposure to side effects (Meidahl et al., 2017).

Adaptive DBS (aDBS) offers to solve many of the limitations of cDBS systems (Arlotti et al., 2016; Meidahl et al., 2017). In this approach, stimulation is delivered in a closed-loop format that allows the system to adapt to the patient's state. Stimulation can be applied only when necessary, thereby reducing side effects while maintaining clinical efficacy. Since the stimulation could adapt to the severity of symptoms, stimulation would always be delivered at the optimal level. Moreover, recent evidence suggests that intermittent stimulation may be more effective at suppressing symptoms than cDBS (Little et al., 2014; Ferleger et al., 2020). ET is a particularly attractive application for this approach since the primary symptom, tremor, manifests itself almost exclusively during movement. This clearly defines the periods when stimulation would be the most beneficial, greatly reducing the complexity of the control problem to be solved. It is worth noting that naming several conventions exist, with adaptive, closed-loop, and responsive DBS having overlapping definitions. In this work we use adaptive DBS as an umbrella term to describe the various ways in which we have automatically adjusted stimulation based on biomarkers of the patient's state

At the start of our study, aDBS had been demonstrated successfully in patients with PD. Several studies have even shown that in some cases aDBS could be more effective than traditional cDBS or randomly applied intermittent stimulation in ameliorating certain symptoms of PD (Little et al., 2013, 2014). However, at the start of our study, there was only one known attempt at developing aDBS for ET. In that study, the authors used motion detected through an EMG system on the patient's arm as a control variable for turning DBS on and off (Yamamoto et al., 2013). This study and the encouraging results in the PD space guided much of our early work.

In this paper we review the development process of aDBS in ET patients carried out at the University of Washington. We begin with an overview of both the hardware and software in the research platform we developed around the Medtronic Activa PC+S. We then outline how we reproduced earlier results and developed a proof-of-concept aDBS system using cortical LFPs as a control signal. We then improved this system by leveraging machine learning and investigating volitional BCI-style control of aDBS. By the end of the study, we had arrived at a clinically translatable, fully implantable aDBS paradigm, accompanied by a largely automated programming process. Consequently, aDBS seems to have now reached the threshold where it could be evaluated as a clinical therapy to improve patients' lives. This however brings with it a plethora of neuro-ethical and practical consideration which we discuss. This review paper is intended to provide a an overview of the development process and preliminary clinical results from start to finish. We hope it will provide a unique viewpoint and present practical context for the ongoing development of aDBS for ET.

## 2. System Integration of the Activa PC+S and Nexus-D Systems

**Figure 1:**
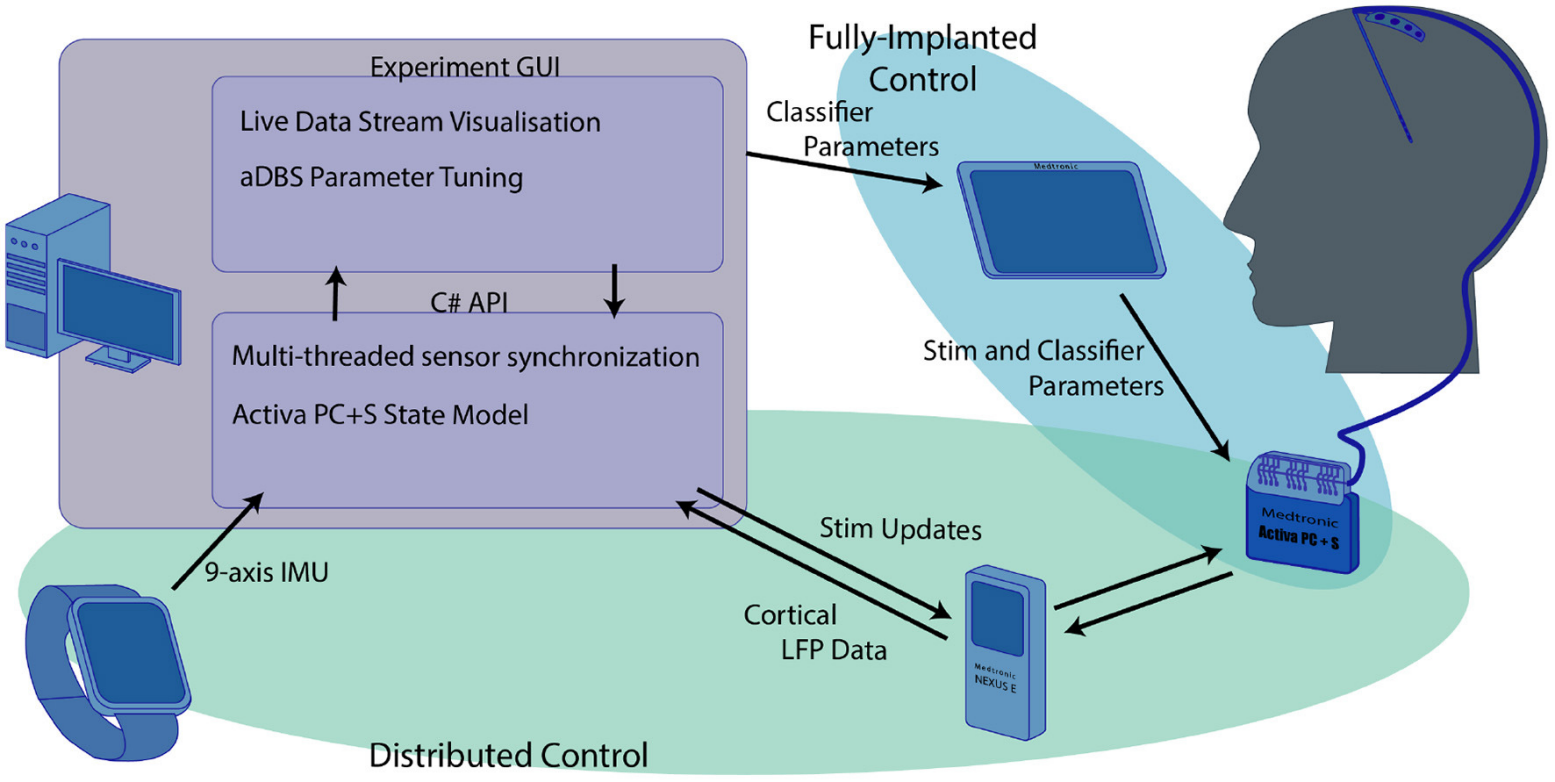
Overview of the research system used for the experiments we describe here. The computer functioned as the central hub that coordinated both the distributed aDBS paradigms and provided a training platform for the fully implanted algorithm. The distributed systems, highlighted in green, used the computer to integrate data from both the smartwatch and real-time cortical LFP streamed through the nexus to drive aDBS stimulation updates. Although the streamed cortical data and IMU smartwatch were used in training the fully implanted classifiers, these classifiers were independent of the main computer once they were transferred to the Activa PC+S using the Medtronic tablet.

The research system we developed was an integration of multiple independent components. As a result, the system required a significant amount of software development. A full schematic of the system is shown in Figure 1. All research was carried out with the approval of the UW IRB and the FDA. Patients provided informed consent before participating in the study.

### 2.1. The Medtronic Activa PC+S and Nexus-D

The central component of the research system was the investigational-use Medtronic Activa PC+S, used with FDA permission under an investigational device exemption (IDE, clinical trial number NCT02443181) (Stanslaski et al., 2012). This device consists of a pulse generator implanted (IPG) in the chest which controls both stimulation and sensing capability. The IPG is connected via a subcutaneous clinical lead extension to the stimulation and sensing electrodes. For this protocol, stimulation was delivered using a clinical standard four-electrode DBS stimulation lead, the Medtronic Model 3387, implanted into the ventral intermediate nucleus of the thalamus (VIM). Sensing was performed utilizing a Medtronic Resume-II four-contact strip electrode placed on the surface of the cortex, spanning the central sulcus, roughly over the hand motor area. This configuration allowed for standard tremor-mitigating stimulation to be delivered to the VIM while also allowing the sensing of cortical local field potentials (LFPs) related to hand motor activity. The IPG supports both cDBS and aDBS. cDBS can be configured using the Medtronic 8,840 clinical programmer. aDBS can be performed either in a distributed fashion with control decisions made outside the device, or in a fully implanted fashion with stimulation decisions made on-board after configuration using the Activa PC+S Sensing Tablet. For distributed control of stimulation, the IPG can be paired via a short-range inductive connection with the Medtronic Nexus-D or Nexus-E telemetry bridges. The choice of sampling frequency for the neural data was largely driven by the hardware specifications. This setup can stream raw LFP data to a desktop computer via a USB connection with a sampling rate of up to 422Hz if streaming from one electrode, or 200 Hz if streaming from two simultaneously. In the work presented here, we used the 422 Hz LFP data streams. We found that the benefit to aDBS control of the addition of VIM data was not worth the loss in sampling rate, since LFP data from the VIM was heavily contaminated by stimulation artifacts. However, data is transmitted in discreet packets every 400ms. In practice, the half-duplex inductive link's bandwidth limitations resulted in a small window for stimulation updates to be transmitted to the PC+S without resulting in streamed neural data loss, so stimulation updates in a distributed algorithm needed to be performed with 400 ms resolution. All neural recordings consist of differential voltage recordings between pairs of electrodes. In this use-case we selected our cortical recordings to utilize pairs of electrodes that lay on opposite sides of the central sulcus. These were identified as the pair with the highest beta-band power while the patient was at rest, determined through a standardized montage-sweep provided by the Activa PC+S instruments. The Activa PC+S can also stream analog estimates of power bands at a sampling frequency of 5Hz, with frequency ranges configured on the proprietary Medtronic Sensing Tablet. When not streaming, the sampling rate of the IPG can be increased up to 800 Hz for raw LFP data, and the resulting LFP or power band data can be downloaded to a USB drive via the Medtronic tablet. In the fully implanted aDBS configuration the IPG uses the analog power band estimates from the attached electrodes in combination with a simple linear classifier to control stimulation. Both the power bands and the weights of the classifier are also configured via the Medtronic Sensing Tablet.

### 2.2. Tremor Sensing and Measurement

To better evaluate the severity of the patient's symptoms and the efficacy of the aDBS paradigm, several modalities of data were collected in parallel to those described above. An Android smartwatch worn on the patient's wrist capable of streaming 9 axis inertial measurement unit (IMU) data (3-axis accelerometer, 3-axis gyroscope, and 3-axis compass) at 100 Hz, was connected to the PC via a Bluetooth connection. This IMU data served as the basis of quantitative and automatic evaluation of tremor. In addition, we asked the patients to perform the tasks from the Fahn-Telosa-Marin tremor rating scale (FTM) (Fahn et al., 1988). Spiral and line drawings were recorded either on paper or through a custom application on a Microsoft Surface tablet (Sonnet et al., 2020). These, along with videos of the patients, were then rated by a panel of three blinded neurologists to obtain an objective, clinically translatable metric to evaluate the efficacy of DBS. For some experiments, we also used the gTec Mobilab to collect EMG from the study participant's tremoring limb using wet gel electrodes as both a trigger for stimulation and as a method of evaluating the effect of aDBS.

### 2.3. Software Development and C# API

The distributed elements of the research platform described above were integrated through the PC using an application development framework written in C# (Herron et al., 2017). This framework utilized a custom developed C# API for generic Nexus-D/Activa PC+S control which enabled simultaneous communication with all sensors and asynchronous dispatch of commands to the Activa PC+S. This C# API was used to develop protocol-specific applications responsible for the collecting and processing data from additional sensors. Multi-threaded coding techniques were used to ensure that sensing, processing, and device communication would not impact the responsiveness of the closed-loop algorithms being investigated. This was further complicated by the fact that the Nexus D and E could not concurrently send data and receive a command, leading to a very narrow timeout window. The API therefore independently maintained an internal model of the Nexus and IPG system states to ensure that all command timings remained in sync. This had additional battery power-saving benefits as the Activa IPG did not need to be queried for its system state. Even with this precise timing capability, the half-duplex nature of the communication hardware and the narrow window for stimulation adjustments to be made before the next data packet needed to be transmitted resulted in a potential delay of 800 ms between the time a biomarker appeared in the patients' brain signals and the time the system could respond by adjusting DBS.

Using this framework, we constructed an experiment control application that enabled rapid development and testing of novel aDBS paradigms. This application, also written in C#, allowed data from any subset of the potential signal sources (neural or wearable) to be streamed simultaneously. Each sensor's data could be visualized in real time and used to control stimulation. Under the hood, each new aDBS paradigm was implemented by editing a single class within the application. This class managed the buffers for all data streams and made the required most recent data available. Once the decision about how to adjust stimulation amplitude was made, the change was passed through another buffer to the Nexus-D API which handled the changing of stimulation. To minimize side effects, stimulation was slowly ramped up, step wise to and from its maximum value. A maximum ramp rate was set for each patient and the software was configured to send individual simulation change commands at the appropriate clock times to manage ramping. This setup allowed development of each new aDBS approach to focus on the meaningful interpretation of biomarkers and stimulation patterns rather than control of individual sensors and timing of stimulation updates.

### 2.4. *Post-hoc* Framework

**Figure 2:**
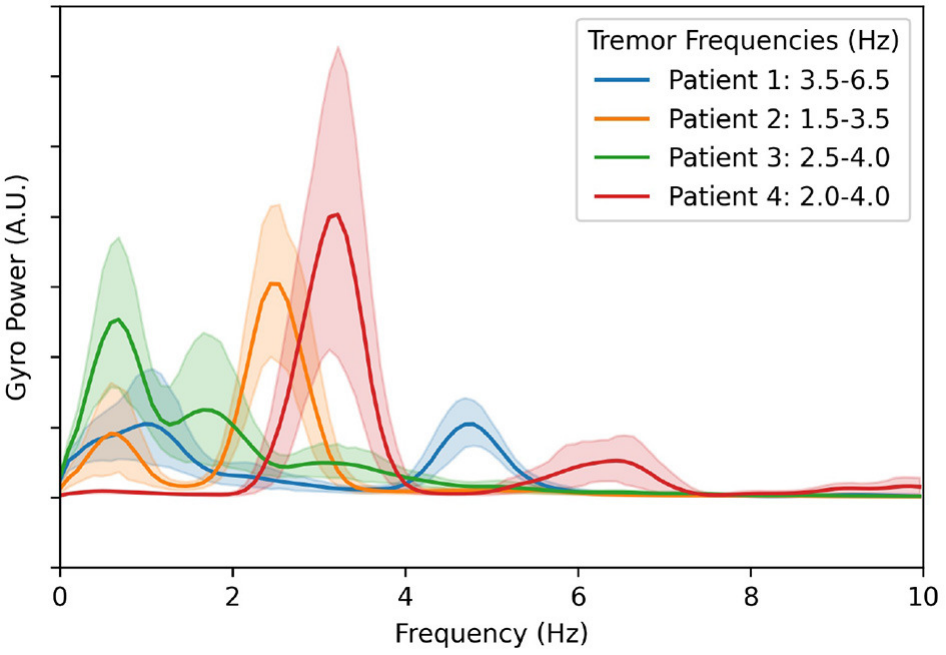
Averaged gyroscope magnitude power spectra from all movement periods during all experiments and calibration sessions with DBS turned off. The first peak in each patient's tremor spectrum above 1.5 Hz is considered to be the patient's peak tremor frequency. The resulting frequency ranges used to estimate tremor severity for each patient are shown in the legend.

To accurately compare the effectiveness of each of the aDBS algorithms discussed here, we use an evaluation of tremor based on the IMU gyroscope data. For this, we calculate the total power in the frequency band that corresponds to each patient's maximum tremor amplitude. The power spectral density along each of the three axes was calculated independently, and then the magnitude was taken for each frequency using the Euclidean distance. The results are plotted in Figure 2. The components below 1.5 Hz are considered normal characteristics of movement, power in this band indicates that the patient is actively moving. The largest peak in the spectrum in the 1.5–8.0 Hz band was determined to be the patient's peak tremor frequency. Power in this band quantifies the amount of tremor the patient is experiencing.

Tremor algorithms were also evaluated based on total power delivered. Since the onboard circuitry of the Activa PC+S uses relatively little power, stimulation is the largest drain on battery power. Since the Activa PC+S was not rechargeable, ensuring that algorithms delivered stimulation effectively with respect to power consumption was important. To calculate total electrical energy delivered (TEED) we used the following metric:


(1) $$\begin{array}{l} TEED=\frac{V^{2}\cdot f\cdot p}{z\cdot \tau }\cdot t \end{array}$$

where *V* is the voltage, *f* is the stimulation frequency, *p* is the pulse width, *z* is the impedance, τ is the duration of the experiment, and *t* is the duration that stimulation is applied at this voltage (Moro et al., 2002; Koss et al., 2005). To make this metric easier to understand, we give values for TEED as the ratio between the TEED by aDBS, and TEED as if cDBS was applied with the same stimulation parameters. For comparison between algorithms, we use both *TEED per second during movement*, and *TEED per second during rest*. Lower TEED during movement with minimal tremor indicates that the stimulation paradigm used was efficient in suppressing tremor. TEED during rest indicates that the algorithm delivered stimulation even when it might not have been necessary. Since suppressing tremor is the prime purpose of aDBS, minor over-stimulation is not considered to be the primary concern. However, if over-stimulation leads to excessive side-effects, or rapidly drains the battery, then it quickly become unacceptable.

At the end of this multi-year study, we had amassed a large collection of longitudinal data. Due to the iterative nature of the development process, the data was not stored in consistent formats and did not always have complete metadata. We therefore developed a python analysis framework to standardize the data formats and enable large-scale longitudinal analysis of all the experiments and data modalities collected. This framework, built around a flexible experiment object, can search a directory tree to discover any sources of potential data and attempt to interpret missing metadata. This resulting dataset can easily be filtered by experiment type or data modalities available. Additionally, any missing or corrupted data resulting from a loss of connectivity or sensor saturation, respectively, was detected and filtered out. This framework has been used to examine the stability of biomarkers over the duration of the study period, as presented in Fraczek et al. (2021), and discussed in Section 4.3. In the context of this work, we used the framework to re-illustrate previously published data.

## 3. Development of aDBS for Tremor

Our effort to develop aDBS for ET proceeded from a technology demonstration study in one patient to a clinically translatable, fully implanted system. Here, we will outline the process by which we developed each of these systems and the most important outcomes that informed the next generation of the work.

### 3.1. Initial Demonstrator

Initial feasibility studies began with the implantation of our first patient in 2015. The goal of this initial work was to demonstrate that system integration was successful and could be used to prototype aDBS paradigms, which had never been done before (Herron et al., 2015, 2016). These initial experiments would validate the system and allow us to correlate the various modalities of data to the patient's state. Using this data, we began the development and verification of neural biomarkers of movement and tremor to enable neural-driven aDBS (Herron, 2016; Herron et al., 2017). To objectively evaluate the effectiveness of any aDBS paradigm, we needed to develop an IMU-based measurement of tremor that could track fast changes in tremor severity. This would then be a test bed that would enable the identification of biomarkers that would not only reliably distinguish times when stimulation was needed but also be robust to changes in stimulation.

**Figure 3:**
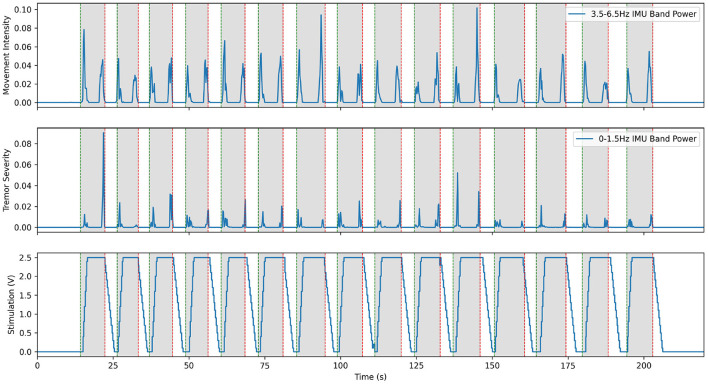
Excerpt from an experiment demonstrating aDBS driven by EMG on the moving arm with Patient 1. The top plot shows the power in the low frequency IMU data, which corresponds to movement. The middle plot shows power in the band of IMU data that corresponds to the patient's peak tremor frequency. The bottom plot shows the amplitude of stimulation over time, with a constant 150 Hz frequency and 90 us pulse width. Note that tremor is very effectively suppressed in this case, with only very short bursts of tremor at the very start and end of movement. This is a re-illustration of the results of Herron et al. (2015).

Hardware validation experiments with our first patient began before the post-operative lesion effect wore off, about 2 weeks after the implantation surgery. During this time, we calibrated the Activa PC+S recording capabilities and fine-tuned the research setup. The lesion effect had disappeared by the third visit, and the difference in tremor between the stim on and stim off states was fully visible in spiral drawing tasks. Taking inspiration from prior literature described in Yamamoto et al. (2013) where EMG was used to drive a DBS system using a re-engineered patient programmer, we first implemented prototype aDBS paradigms driven by EMG and IMU signals. Each of these devices was used to monitor the patient and detect whether they were actively moving. When movement was detected, stimulation was rapidly ramped up to the therapeutic threshold and maintained until the patient returned to rest. A representative trial for the EMG system is shown in Figure 3. These trials consisted of a comparison of the relevant data during repeated rest, movement, and imagined movement trials. During rest, the patient was asked to simply sit in the chair, while all sensors recorded data to use as a baseline. During movement, they were prompted to raise their hand (at the time of the green vertical lines) and hold it out in front of themselves, until prompted to return to rest (at the time of the red vertical lines). This movement was found to reliably elicit tremor for this patient. Prompt intervals of various lengths, were interleaved so that multiple comparisons could be collected quickly. A similar approach was used for imagined movements, but instead of moving, the patient was asked to instead imagine performing the same movement. This prompt paradigm was used as a template for many of the later experiments through the study. Data collected during these trials allowed us to verify that data was correctly streaming to the central control desktop. Analysis of the IMU data allowed us to develop a metric for tremor severity, described below and shown in the second plot below, which could be computed in near real time. We found this metric correlated to the tremor observed in the patient, based on the FTM scale, while reducing the movement onset and offset artifacts (Herron et al., 2017). By comparing the neural data obtained during these trials we tested whether our system was able to detect beta band desynchronization both during overt and imagined movement. Moreover, these changes were apparent even during stimulation, despite the dramatic changes in the frequency spectrum observed during DBS. Throughout this initial process, we conducted interviews with the patients to assess their level of comfort and gain a greater understanding of the patient experience.


(2) $$\begin{array}{l} \text{Tremor Severity}=\frac{{\left(\text{IMU Tremor Band Power}\right)}^{2}}{\text{IMU Total Power}} \end{array}$$

This system fulfilled its primary goal as a technology demonstrator. aDBS triggered by movement, particularly in the case of EMG, was successful in suppressing tremor while delivering less total stimulation. With an average delay to max level stimulation of 2.40 ± 0.33 s, this resulted in stimulation delivered 76.6% of the time the patient was moving and 15.3% of the time the patient was at rest. This resulted in tremor severity (per Equation 2) during movement of 0.277 compared to 1.296 during no stimulation and 0.6473 during cDBS trials conducted with the same patient during the same session. aDBS driven directly by tremor severity interpreted from IMU data was less successful, due to feedback causing the stimulation to fluctuate wildly. Beta band desynchronization was shown to be reliably identifiable with the hardware available, and therefore a potential control variable for future aDBS systems triggering off of movement-related biomarkers. Although this initial work showed the potential of our system as an investigational device and the promise of aDBS for ET, it also highlighted many of the challenges that would need to be resolved over the rest of the study. Conversations with the patients exposed their reticence to undergo battery replacement surgery, thereby highlighting the importance of conserving battery power. Since streaming neural data used approximately 10 times as much battery as normal operation, experimental sessions were kept succinct and avoided unnecessarily draining the patient's battery. Moreover, using aDBS to minimize the energy usage of stimulation would be an important consideration throughout the rest of the project. The delays inherent in the distributed Activa PC+S system made the control scheme for distributed aDBS difficult to implement. We often observed transient periods of significant tremor at the onset of movement, before the aDBS control caught up and turned-on stimulation. Solving this issue would be one of the main targets we would pursue.

### 3.2. Distributed BCI Control

**Figure 4:**
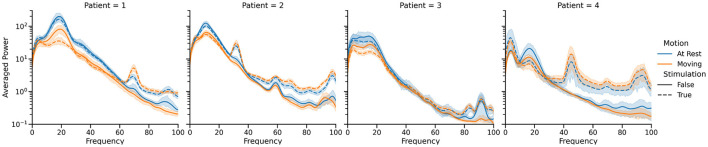
Summary of the power spectral differences observed in cortical LFP recordings between the four states of primary interest for each of the four patients who participated in our study. Only single source recordings, performed at the hardware-specified 422 Hz, were used in this visualization, and movement periods were identified by manual inspection of the IMU data. Note the difference in spectral characteristics between the stim off (solid lines) and stim on (dotted lines) cases, as well as the drop in 12–30 Hz band power during movement (orange) compared to rest (blue). These spectra vary significantly between patients, necessitating training of classifiers for each patient individually.

Informed by these initial results, we endeavored to build a neural driven, BCI aDBS system (Herron et al., 2015, 2017; Houston et al., 2017; Castaño-Candamil et al., 2020). A system like this, driven by the well-documented beta band desynchronization phenomenon, would allow stimulation to only be delivered when necessary during movement (Pfurtscheller and Aranibar, 1977; Toro et al., 1994; Unterweger et al., 2020). Since the Activa PC+S is capable of using cortical LFP power bands to control stimulation, validating beta desynchronization driven aDBS in a distributed fashion, would pave the way for fully implanted aDBS systems. As a further extension to this cortically driven aDBS, we also endeavored to build a volitional aDBS system. This approach offered to make aDBS more flexible and applicable to more diseases by handing control of the stimulation directly back to the patient. The idea was that although controlling stimulation would take conscious effort initially, repeated training and daily use would allow the patient to develop automatic, almost subconscious control of the stimulation. As indicated by our conversations with the patients, this also had the potential to greatly improve the patient experience by increasing the sensation of agency and strengthening the identification of the device as a part of themselves (Figure 4) (Brown et al., 2016; Herron et al., 2017).

**Figure 5:**
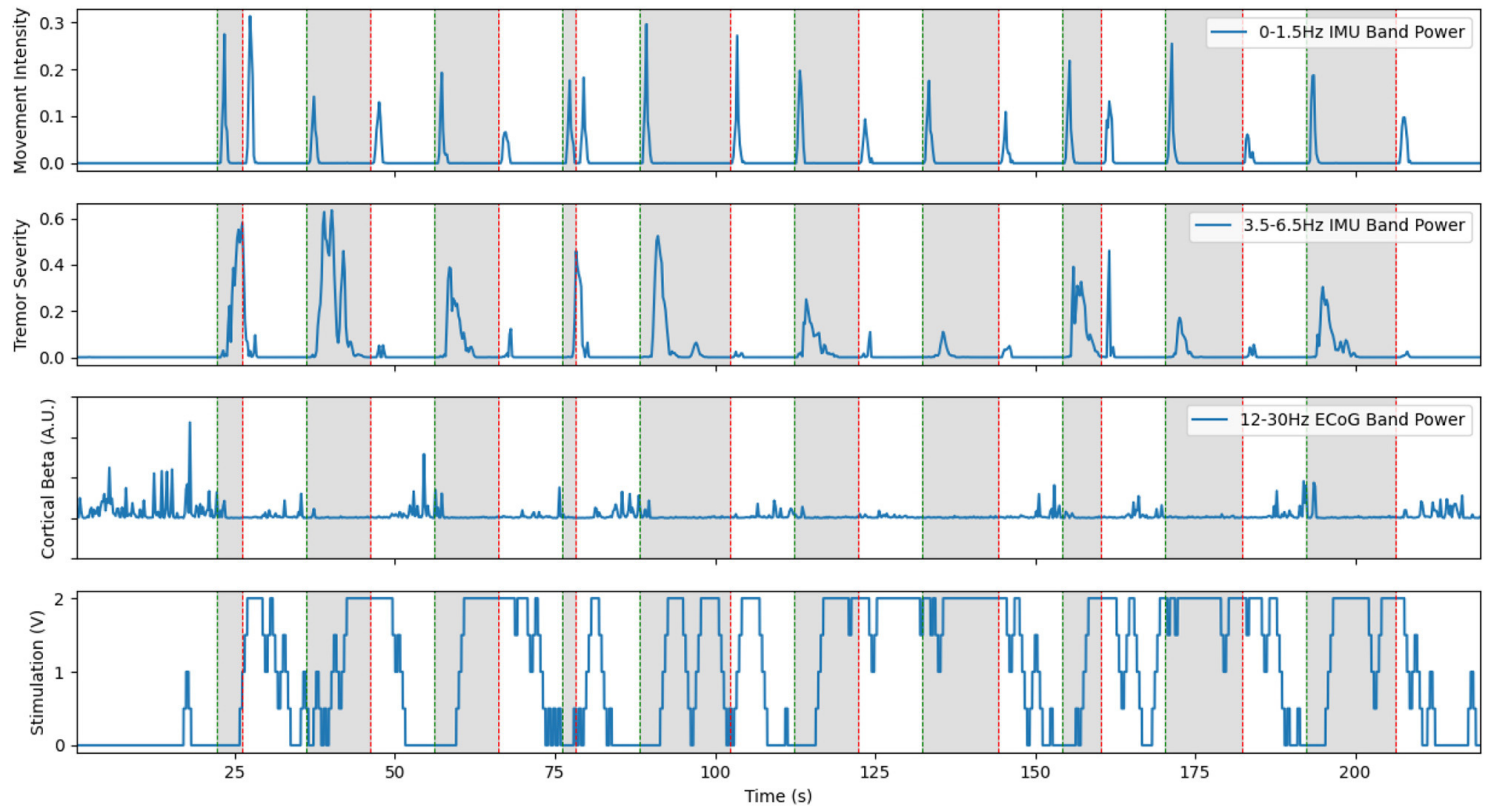
Representative example of cortical LFP driven aDBS using the beta desynchronization classifier approach with Patient 1. The inclusion of a separate classifier for the on and off states allows stimulation to stay on through the duration of prompted movements, shown in gray. However, large delays in this distributed system still led to large burst of tremor near the start of movement. Limited resolution of the LFP recordings occasionally lead to the classifier incorrectly switching on or off, as seen at 15 s and during the prompted movement at 100 s. This is a re-illustration of the results in Houston et al. (2017).

The method we chose to detect movement was the well documented phenomenon of event-related-desynchronization in the beta band (12–30 Hz). This approach consisted of training linear discriminant classifiers to detect significant drops in power in the beta band that corresponded to movement for each patient. Since the application of high-frequency DBS significantly altered the power spectra visible on cortical recordings, two classifiers were trained in parallel, one with DBS off and one with DBS on. Classifier training used the prompted movement task described above. Average power spectra were computed in each of the four states for that patient on that day. A weight was assigned to each frequency bin, based on how much the power in that bin changed between the rest and prompted movement states for each stimulation state. Once the classifiers were trained, the adaptive DBS algorithm proceeded as follows. Starting in the stim off state, the off classifier listened to the neural data stream, calculated power spectra using Welch's method with a Hann window and normalized by the average and standard deviation of the classifier training data. We then took the dot product of this power spectrum with the classifier weights and fed it into a logistic regression function. When this result crossed a pre-set threshold, indicating the onset of volitional movement, stimulation was ramped up to its maximum clinically permitted value over the course of a few seconds, and the system switched to using the stim-on classifier. Since the ramping of stimulation is known to lead to the greatest number of side effects, the ramp rate was carefully tuned to be the fastest pre-set possible ramp rate that was tolerable for the patient. When the stim-on classifier detected that the beta band power had risen back up to levels indicating rest, stim was ramped back down and the system switched back to the stim-off classifier. The progression of cortical beta is shown in the third row of Figure 5. The thresholds were tuned for stimulation sensitivity, as reliably delivering stimulation during movement was considered more important than reliably turning stimulation off when at rest.

The neural BCI approach resulted in a system that could control the delivery of stimulation with a sensitivity of 90 and 100% for the prompted movement and FTM drawing tasks, respectively (Houston et al., 2018). For this trial, stimulation was delivered 64.8% of the time the patient was moving and 29.0% of the time the patient was at rest. Tremor severity (per Equation 2) during movement was 3.750 compared to 0.4338 during no stimulation and 8.271 during cDBS trials conducted with the same patient during the same session. However, over all patients and all sessions, we found a 46.0% average improvement in clinical FTM scores over the no stimulation condition, compared to a 42% average improvement during cDBS. Although the difference between each of these and the stimulation off state was statistically significant, the difference between the two stimulation paradigms was not. A representative trial of this aDBS paradigm is shown in Figure 5. Although the system was always able to identify movement periods in this excerpt, the identification was often delayed and noisy. This was in large part due to the comparatively low spatial and temporal resolution of the cortical strips. Only four electrodes were available, and only a single pair could be used at a time to provide the differential recordings required for the system. The strip was placed during the implantation surgery and could not be adjusted afterwards. This meant that any imperfections in the initial placement and shifts over time left the electrode not in position to optimally observe beta band desynchronization. Moreover, the delay between the onset of movement and the onset of stimulation was 1.5 s on average. For the trial shown above, we observed an average delay to the clinically effective level of stimulation of 3.35±1.50 s. In certain cases, this delay time could reach up to 5 s. This was due to the transmission delays inherent in the system architecture, the extra time required to compute power spectra, and the limitations of the ramp rate. These confounding effects can be clearly seen in Figure 5 as stimulation starts well into the gray prompted movement periods (bottom row), leading to a large burst of tremor before stimulation becomes effective (second row). However, once stimulation did ramp up to clinical levels, the tremor was effectively suppressed.

In a similar vein, we conducted a study in collaboration with the University of Freiburg to investigate distributed aDBS in a way that could more smoothly adapt stimulation and better adapt to the patient state (Castaño-Candamil et al., 2020). This approach used bollinger bands to perform local estimates of high and low tremor states to dynamically drive stimulation, which allowed the system to more robustly respond to movement and non-movement states in a variety of tasks without the need for re-training. Although this work provided a more robust method of driving aDBS that led to greater power savings than the simpler method presented above, the more advanced calculations required meant it could not be implemented in a fully implantable state. However, it remains a promising avenue to explore as the hardware available improves.

### 3.3. Fully Implanted Adaptive DBS

**Figure 6:**
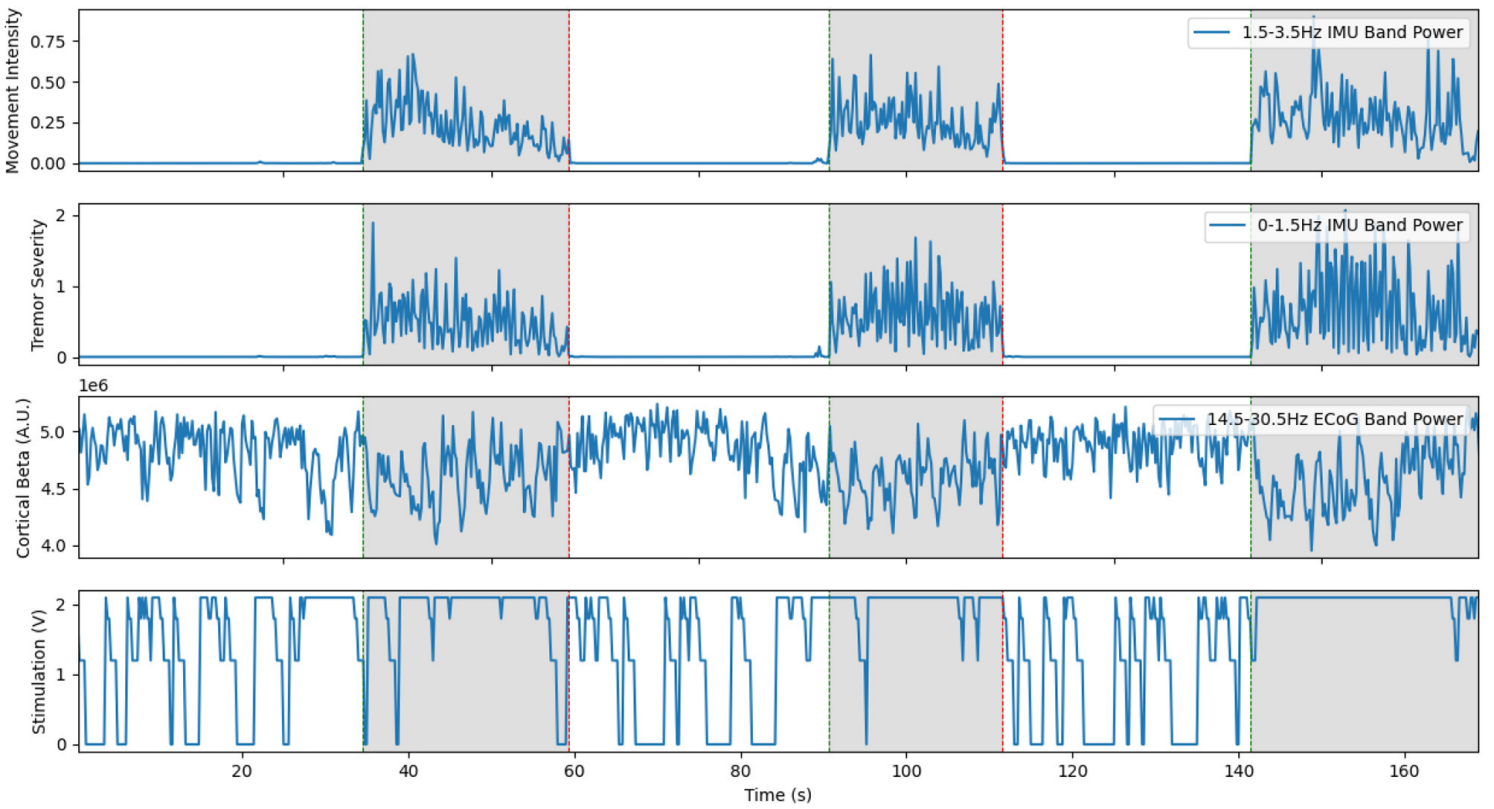
Representative trial of the fully implanted aDBS system with Patient 2. Since the classifier is strongly biased towards having stimulation on, the stimulator turns on even during rest. Unlike the previous examples, the cortical beta shown here is the power band estimate computed online by the Activa PC+S during the experiment. Although this is more noisy than the offline spectra, the system was able to respond very quickly to the onset of movement and turn on stimulation. This is a re-illustration of the results in Ferleger et al. (2020).

The fully implanted system was the culmination of all the work performed and the first potentially clinically translatable system (Ferleger et al., 2020). As identified in previous work, the time delays inherent in the distributed architecture were a major source of aDBS paradigm design difficulties. Since the aDBS algorithm implemented in this case would function entirely within the IPG, these communication delays would be massively reduced. Additionally, there would be no constraints placed on the patient in terms of additional wearable hardware. As a result, IMU data from the android smartwatch and the computational power of a desktop PC could only be used in the training process. The real time updating of stimulation would have to rely entirely on the capabilities of the IPG. Moreover, the resultant system should be easily adaptable to new patients to reduce the large time commitment from both patient and clinician required to tune stimulation parameters. By automating the programming methods used to develop the initial aDBS system, patients could benefit from aDBS without requiring the prolonged manual tuning of the classifier necessary in previous versions (Figure 6).

Classifier training proceeded in a semi-automated fashion leveraging the convenience of a desktop PC to determine a classifier that could be used entirely within the capabilities of the implanted device. Due to hardware limitations, there was no explicit way to pass stimulation state as a parameter to the onboard mechanism. To overcome this, classifiers were trained for each of the four possible states the patient and stimulation system could find themselves in: Stim off, rest; stim on, rest; stim off, movement; and stim on, movement. Thirty seconds of data were collected for each state, with optional repetition of selected tasks. For each of the patients, data from these individual classifiers was combined, which resulted in a classifier that used the power in the band near the stimulation frequency to determine the stimulation state, and the power in the beta band (usually 12.0–28) to distinguish whether the patient was moving or not. Again, since unnecessary stimulation was considered less of an issue that missed stimulation, the classifier was strongly biased towards favoring the stimulated state. This classifier was then uploaded to the IPG and used to switch stimulation on and off. Total time to train this system was under 20 min for each patient. Evaluation of this system was performed in a manner similar to previous experiments, using both tremor suppression calculated from IMU data and total electrical energy delivered.

This system demonstrated the advantages of a fully embedded aDBS system. Although classification had to be performed on simpler hardware, it maintained high levels of effectiveness. The system had a sensitivity of 91.8%, and a false positive rate of 28.7% (Ferleger et al., 2020). Due to classifier bias, and the limited resolution available in this implanted configuration, the classifier often interpreted temporary drops in beta power as movement. Tremor was suppressed as well or even better than cDBS. Overall, the dramatic reduction in control delays seem to outweigh limitation on the complexity of the classifier. Additionally, the ability of this system to be deployed to patients without the need for a tether to external hardware cannot be overstated. This is the first reported, full-translatable, aDBS system for ET. In related work published by our the group at the University of Florida, the value of embedded closed-loop for the treatment of ET has been further demonstrated upon in an expanded group of patients over a time period of several months (Opri et al., 2020). This study used similar cortical biomarkers, but achieved much higher specificity, potentially due to a more sophisticated paradigm for training their neural classifiers. Our approach for the classifier was based on a large block structure. We collected data during movement and rest, with stimulation both on and off. The classifier was then trained by comparing the power spectra between these four conditions. Conversely, the UF team used data from a prompted movement task to train their classifiers. It seems that these repeated small samples were better able to generalize to the tested behavior, potentially by capturing the transitions between states. Additionally, motivated by clinical considerations, we biased our classifiers towards avoiding false negatives. This resulted in our system having a higher sensitivity than it would otherwise by compromising specificity, although the prioritization of therapy over power savings resulted in stimulation turning on when not needed. There is still much that future studies could do to improve performance with newer hardware. However, this system showed better performance in TEED and greater tremor suppression than cDBS. Although more study is necessary to understand the source of this improved performance, it is an encouraging sign that aDBS systems could improve the lives of patients even more than cDBS systems when deployed in clinical practice.

## 4. Discussion

Over the course of this study, we made several important advancements. We demonstrated the feasibility of aDBS for ET using cortical LFPs as a control signal. We then developed preliminary machine-learning driven BCI and volitional control systems. These elements were then put together to create a fully implantable, clinically translatable aDBS system for ET. Over the course of this process, we came to many conclusions that we hope will be helpful to future generations of aDBS for ET. Here we outline some of the most important conclusions that we came to over the course of this study, both practical and theoretical. We also provide our outlook on what the key hardware and ethical challenges that need to be solved in aDBS for ET.

### 4.1. Remaining Work

One of the most prevalent issues that we ran into was the control delay inherent in the distributed system. A large part of this delay can be attributed to the hardware limitations of the Activa PC+S. However, any distributed system will necessarily have larger delays due to the increased communication distance over a fully implanted one. Conversely, a distributed system will have much higher computational power and flexibility than a fully implanted one. As we demonstrated in our embedded aDBS experiments, the tradeoffs between these two types of systems can be minimized by utilizing a hybrid approach. By training the system in a distributed fashion, we can utilize the full computational power that larger hardware offers to adapt stimulation in a high dimensional parameter space. By using this system to set the parameters on a simpler, fully-embedded classifier, we can retain the fast response times of a fully embedded system. Future studies will endeavor to further tighten and automate this two-stage control loop. It is likely that the classifier used in the implanted system will not remain effective for long periods of time as the patient's state and medications change. Multi-modal monitoring of the patient's symptoms, with simultaneous streaming of neural, IMU, and even video data, would detect when these changes occur. The system would then either prompt the patient to re-train the classifier or substitute in a previously trained classifier that would better suit the patient's current state.

As has been well documented; side effects of stimulation, especially paresthesia, are often exacerbated while stimulation is being ramped up. To ensure the comfort of our patients, we always set a maximum ramp rate for each patient that did not induce intolerable side effects. Several studies have noted the nuances of stimulation ramp rates but to our knowledge no conclusive best practices have been established (Petrucci et al., 2021). In our patients, we noticed that the maximum tolerable ramp rate differed drastically. Further studies will be required to better understand this phenomenon. With a better understanding of the nuances of ramp rates, stimulation could be applied in a way to circumvent paresthesia while still allowing for fast control of DBS.

Due to the communication delays discussed above, we often observed large bursts of transient tremor at the onset of movement. Limitations on the ramp rate necessary for patient comfort mean that the delay between the need for stimulation and when stimulation reached clinically effective levels was even longer. Future studies should therefore investigate aDBS systems with different levels of minimal and maximal stimulation. Instead of switching stimulation between the on and off states, we would instead switch between high and low amplitude stimulation states. The low voltage state would be set low enough to not be noticeable for the patient, and the high level would be at the clinically effective threshold. Since the difference in voltage between states would be smaller, this would enable the system to respond faster without inducing ramping side effects.

All our work has focused on changing the amplitude of the delivered stimulation. There is evidence showing that stimulation amplitude offers the most control of any single parameter adjustment (Cooper et al., 2008). However, DBS efficacy is highly dependent on the other two tunable parameters: pulse width and stimulation frequency. Stimulation frequency is of particular importance according to theories that tremor may be caused by excessive coupling between oscillatory activity in different regions of the brain (Raethjen and Deuschl, 2012; Helmich et al., 2013; Filip et al., 2016). If this is the case, adjusting stimulation frequency could reveal stimulation frequencies that both improve and worsen tremor. The existence of multiple harmonic stimulation frequencies that have similar therapeutic effects would be a strong confirmation of this phenomenon. As a result, the effect of stimulation frequency on DBS should be investigated both for the sake of improving clinical effectiveness and for the potential of a greater understanding of the pathology of ET.

Another aspect of aDBS that has been shown to be important is the exact methodology of training the neural data classifier. This is highlighted by the comparison between our results and those recently published from the University of Florida (Opri et al., 2020). It is clear that the training data and paradigm must be designed with the ability to translate into a more naturalistic context in mind. Their study achieved higher sensitivity, specificity, and overall accuracy. When classifiers such as these are designed, we believe it is important to take the patient experience and clinical practice into account. False negatives, lack of stimulation when it is needed, are more detrimental to the patient than false positives, stimulation even when it is not needed. For this reason, it is important that classifiers are optimized primarily for sensitivity instead of just overall accuracy. These considerations will help ensure that aDBS is best optimized for the needs of the patient.

### 4.2. Alternative Approaches

A potential direction of research that we did not fully explore in our study is the potential to smoothly adapt the levels of stimulation to the severity of symptoms. Systems like this could follow a similar approach to the beta thermostat approach demonstrated in the PD literature (Qasim et al., 2016; Swann et al., 2018), adjusting stimulation to even out therapy when provided in conjunction with medication. We performed proof-of-concept distributed studies using bollinger bands to drive 'graded-DBS' (Castaño-Candamil et al., 2020). This work showed promise in handling the non-stationary dynamics and adapting the stimulation algorithms in a patient-specific way. However, hardware limitations prevented us from implementing this approach in a fully implanted context. This approach would allow the aDBS system to adapt stimulation levels more precisely to the changes in patient.

One of the great remaining hurdles in the development of aDBS for ET and treatments for ET in general is the limited understanding of the pathology of the disease. Although progress has been made recently suggesting the involvement of multiple network components and the central role of the cerebellum many unanswered questions remain (Raethjen and Deuschl, 2012; Filip et al., 2016; Ibrahim et al., 2020; Pan et al., 2020). With this growing evidence for the role of the cerebellum, it will be important to identify whether the cerebellum is the sole generator of pathological oscillations, or do further changes need to happen for resonant frequencies to arise. Moreover, it is unclear whether pathological changes occur in other brain region that facilitate the propagation of tremor oscillations. An improved understanding of the pathology of ET could lead to new stimulation targets and stimulation paradigms that better counteract the symptoms of ET, or even pharmacological treatments that directly target the underlying pathological changes. Due to the acute control possible in aDBS systems, this research will be able to help answer many of these important questions.

aDBS for ET lacks the sort of robust neural biomarkers that are directly correlated to symptoms. In PD, for example, abnormally long bursts of STN beta activity has been shown to be directly correlated to symptoms, while a sharp peak in the gamma band has been shown to be directly correlated to stimulation-induced dyskinesia, resulting in a perfect control signal for ramping DBS amplitude up and down (Whitmer et al., 2012; Little et al., 2013; Swann et al., 2018). The ET DBS systems described here must rely on proxy biomarkers such as beta band desynchronization as a measure of movement, which is correlated with symptoms. Since this biomarker imperfectly follows symptoms, there is a hard cap on how close to optimal performance our systems can come. Thankfully future aDBS research is well positioned to begin unraveling these questions. Recent work has shown evidence that there may be alternative biomarkers of movement visible in VIM LFPs (Opri et al., 2016). The potential of this VIM approach was further demonstrated by the researchers at Oxford, who developed a VIM based aDBS paradigm using extremely detailed neural recordings (He et al., 2021). We look forward to seeing how this work develops the field, as VIM aDBS would remove the need to implant the additional cortical strip required for our approach. If a direct biomarker of tremor severity, identifiable both during and off stimulation, could be found, the aDBS could be driven exactly as needed. Further verification and development of both these and cortical biomarkers of tremor is essential for robust aDBS systems for ET. The presence of multiple simultaneously computable biomarkers would allow for cross-validation and increased robustness.

Future studies of aDBS with larger numbers of patients will also be capable of investigating the variations observed between patients. Recent work is increasingly suggesting that ET is not a single disorder, but rather a family of related disorders that need to be treated slightly differently (Soto and Fasano, 2020). This is also supported by the wide variation in effective stimulation settings observed even in our relatively small cohort of patients. For most ET patients, the recommended stimulation frequency is close to 140 Hz, but for one of our patients we found the most effective stimulation occurred near frequencies of about 90 Hz. A survey of optimal stimulation parameters determined in an automated way, matched with neural recordings on and off stimulation would be a promising avenue to investigate these differences. In this context, aDBS is firmly in the regime of personalized medicine. Future aDBS applications should retain the focus on tuning stimulation individually to the needs of each specific patient. Broad generalization is useful only in so far as it simplifies the training process of each patients individualized aDBS paradigm and highlights the nuances of each patient's needs.

One of the developments that could most dramatically push aDBS for ET forward is a well-verified, explanatory model of ET. Specifically, such a model should explain at a high level the interactions between brain areas that give rise to pathological ET tremor oscillations. Though one such promising model has recently been proposed, more work is needed to verify this model and determine how it can be fit to patient data (Yousif et al., 2017; Duchet et al., 2020). This modeling effort should proceed in conjunction with the imaging-based modeling efforts (Dembek et al., 2017; Al-Fatly et al., 2019; Middlebrooks et al., 2021b). As these models move towards more predictive power, they will would allow for more insight during the process of selecting the implant site and tuning aDBS parameters for new patients (Middlebrooks et al., 2021a). Neural and biophysical recordings of patient state could be used to cluster the patient with other patients that display similar symptoms, with the expectation that similar stimulation would be similarly effective for patients within a cluster. This could dramatically reduce the number of parameters sets that need to be tested to find an effective stimulation paradigm. When repeated with multiple patients this would result in a map of ET disease states and related diseases. Even once DBS parameters are set, it is likely that over the course of the patient's daily life, optimal stimulation parameters will change. As the patient takes medicine, for example, the stimulation amplitude and frequency change. A reliable model could then be used to help inform automatic switching of stimulation parameters as the patient's state changes. Fundamentally, a good enough model could provide insight into the pathology of the disease and aid the search for ET treatments that target the underlying cause rather than just treat symptoms.

### 4.3. Challenges of Clinical Translatability

As has been noted in several reviews in this field, there is a large gap between the experimental demonstration of aDBS and a clinically translatable treatment (Arlotti et al., 2016; Meidahl et al., 2017). We took part in collaborations to address some of these challenges with our development of automated tools for optimization of DBS and aDBS paradigms (Haddock et al., 2018). However, several challenges remain.

One of the largest challenges to the clinical translatability of the neural-driven aDBS systems we developed over the course of this study is the availability of on-label cortical strips. These are a required component for the implementation of aDBS systems with that use movement intention sensed from cortex as a control parameter. As implied by our BCI control work, these could also be used in a volitional fashion to seamlessly offer the patient a greater degree of control over stimulation. Recent work along with our own analysis have shown that cortical electrodes such as these retain a high signal to noise ratio for years after implantation (Nurse et al., 2017; Fraczek et al., 2021). This has been further confirmed by the group at UF, who have shown that aDBS driven by cortical strips is robust over several months (Opri et al., 2020). Moreover, in the context of our study we observed no adverse effects as a result of the implantation of the cortical strips, which may motivate work pursuing their clinical validation as a safe extension to existing DBS systems.

In the fully implanted aDBS system we describe above, training of the implanted algorithm proceeds in a distributed fashion. In our experiments, we train and test the classifiers within a reasonably short time scale of less than a few hours. However, this begs the question of how often these sorts of classifiers will need to be updated. If training need only be repeated once every few months, then a system like this could be tuned during routine clinical visits. Thanks to the automated nature of the system, this could be carried out by a trained technician instead of a neurologist. However, if the algorithm requires updating on a near daily basis, then any clinically translatable approach would need to be deployable to the patient's home. Both the algorithm update itself and detection of the ineffectiveness of the current algorithm would have to be fully automated. It is also not clear how well the control variables used in our experiments would translate to daily activities. In either case, more work will be required to evaluate the robustness of aDBS paradigms developed in this way.

Finally, there are a number of ethical considerations that arise as aDBS systems are translated into common clinical practice. We have discussed a number of these elsewhere, but we provide a short review here (Brown et al., 2016). Concerns have been raised about the potential for stimulation to cause shifts in the user's perception of selfhood and agency e.g., in cases where stimulation causes behavioral changes as a side effect (Klein et al., 2015). aDBS might 1 day mitigate and manage these side effects. Questions remain, however, about how users will interact with more robust aDBS systems; how those interactions impact clinical outcomes and quality of life (Brown, 2020). It is not clear, for example, how much control users will want over stimulation parameters, or how involved they want to be in aDBS algorithm training, or how different algorithms will impact user experience. To investigate these questions, a neuroethicist on our team (TB) lead a series of longitudinal, semi-structured phenomenological interviews with each patient the goal of which were to give patients the opportunity to describe using the experimental aDBS platform (Brown et al., 2016). A final analysis of these interviews is underway.

### 4.4. Hardware and Future Systems Outlook

Next generation systems which have been developed and are on the horizon offer significant improvements over the Activa PC+S system.

The Medtronic Summit RC+S offers many of the same capabilities as the Activa PC+S but with greatly improved specifications (Stanslaski et al., 2018). The Summit system can record at 1 kHz, which enables use of gamma band activity (50–100 Hz) to inform aDBS decisions without excessive contamination from Nyquist noise. This is coupled with more advanced, onboard digital spectral power estimation hardware that will provide better accuracy and resolution than the analog system available in the Activa system. The Summit also supports a much more intricate state table for switching stimulation parameters in a fully embedded fashion. This device is already being used to perform aDBS research at several locations (Petrucci et al., 2020; Johnson et al., 2021). The increased capabilities of the Summit RC+S will allow the embedded system to detect biomarkers more reliably and respond with stimulation changes faster and more precisely. Since the device is also re-chargeable the worry about battery conservation is greatly reduced. Battery intensive experiments and streaming of neural data can now be done in the patient's home without excessively accelerating the need for a battery replacement surgery. Such day-to-day monitoring will allow for a more nuanced understanding of both DBS and the disease being treated.

Another upcoming system is the Medtronic Percept (Goyal et al., 2021). This system does not include cortical electrodes, but still offers the ability to record from the implantation target. Raw LFP data can be streamed out to a programming tablet in a manner similar to the Activa PC+S. Alternatively, chronic average band powers on pre-set power bands can be recorded to the on-board memory of the device, and then later downloaded for analysis. Although this device offers lower resolution data than the Summit, it is labeled for clinical treatment. This means that chronic sensing during stimulation could soon enter more regular clinical practice as a diagnostic tool that can aid clinicians in adjusting stimulation parameters. As a side effect, future studies could leverage this chronic data to better understand the pathology of ET and as a jumping-off point for next generation aDBS paradigms. When combined with wearable sensors to monitor the patient's activity, this could prove an invaluable tool for adapting aDBS into daily life.

## 5. Conclusions

aDBS for ET remains a growing area of investigation. Since the start of this study, the field has developed from a single demonstrative case study to a clinically translatable approach. We have developed aDBS for ET from its initial state to a fully implantable system that could be adapted to clinical practice. This fully implantable system is able to suppress tremor more effectively than cDBS while delivering less total stimulation. Despite this, numerous avenues for advancement remain. The longitudinal efficacy of fully-implanted aDBS algorithms have not been tested in a chronic, at-home environment. All work to date has trained and tested the classifiers within the space of a day, so it is likely that transitioning to a chronic setup will require the development of automated tools that would allow the patient to re-train the aDBS algorithm on a regular basis with minimal input from a clinician. Any clinical translation of this work will depend on the availability of safe, reliable, on-label cortical electrodes. We look forward to the life-changing work that will be done in this space as new hardware, techniques, and understanding becomes available.

## Author Contributions

TF prepared the figures and wrote the manuscript. BF helped with figure preparation. TB provided input on neuro-ethical considerations and drafting the relevant sections of the manuscript. BF, MT, AH, BH, and JH performed the experiments reviewed here. JO, AK, JH, and HC provided oversight of the research. All authors reviewed the final manuscript.

## Funding

This work was supported by a donation of devices and funds from Medtronic. Reviewed work also includes funding from the following sources: the Department of Defense through the National Defense and Engineering Graduate Fellowship (DoD NDSEG), the National Science Foundation Graduate Reseach Fellowship Program (NSF GRFP), the National Institutes of Health (NIH) award R01-NS065186, BrainLinks-BrainTools Cluster of Excellence funded by the German Research Foundation (DFG, grant no. EXC1086), the Federal Ministry of Education and Research (BMBF, grant no. 16SV8012), and by Award Number EEC-1028725 from the National Science Foundation for the Center for Sensorimotor Neural Engineering, now known as the Center for Neurotechnology.

## Author Disclaimer

The content is solely the responsibility of the authors and does not necessarily represent the official views of any funding source.

## Conflict of Interest

The authors declare that the research was conducted in the absence of any commercial or financial relationships that could be construed as a potential conflict of interest. The authors declare that this study received hardware and funding from Medtronic. Medtronic was not involved in the study design, collection, analysis, interpretation of data, the writing of this article or the decision to submit it for publication.

## Publisher's Note

All claims expressed in this article are solely those of the authors and do not necessarily represent those of their affiliated organizations, or those of the publisher, the editors and the reviewers. Any product that may be evaluated in this article, or claim that may be made by its manufacturer, is not guaranteed or endorsed by the publisher.
